# tDCS Modulatory Effect on Reading Processes: A Review of Studies on Typical Readers and Individuals With Dyslexia

**DOI:** 10.3389/fnbeh.2018.00162

**Published:** 2018-07-31

**Authors:** Alice Cancer, Alessandro Antonietti

**Affiliations:** Department of Psychology, Università Cattolica del Sacro Cuore, Milan, Italy

**Keywords:** tDCS, neuromodulation, reading, dyslexia, intervention

## Abstract

The possibility to use non-invasive brain stimulation to modulate reading performance in individuals with developmental dyslexia (DD) has been recently explored by few empirical investigations. The present systematic review includes nine studies which have employed transcranial direct current stimulation (tDCS) aiming at improving reading abilities in both typical readers and individuals with DD. Anodal tDCS over the left temporo-parietal cortex—a region which is typically involved in phonological and orthographic processing during reading tasks and underactive in individuals with DD—was the most frequently used montage. The majority of studies employing such stimulation protocol showed significant improvement in differential reading subprocesses. More precisely, word decoding was improved in adult readers, whereas non-word and low-frequency word reading in younger individuals. Furthermore, tDCS was found to be specifically effective in poor readers and individuals with DD rather than typical readers, in spite of the specific brain region targeted by the stimulation; Left frontal, left temporo-parietal, and right cerebellar tDCS failed to modulate reading in already proficient readers. Overall, tDCS appears to be a promising remedial tool for reading difficulties, even when applied to younger populations with reading problems. Further empirical evidence is needed to confirm the potential of neuromodulation as a successful intervention method for DD.

## Introduction

Developmental dyslexia (DD) is a neuropsychological disorder affecting the ability of reading. More precisely, the behavioral manifestations of DD include an inaccurate and/or slow decoding of written language, resulting in a hesitant and effortful reading. Such difficulties are not the consequence of intellectual deficit, sensory dysfunction, socioeconomic disadvantage, or lack of educational opportunities (Snowling and Hulme, 2012; American Psychiatric Association, 2013).

The majority of the intervention methods for DD which have been studied to be effective in overcoming dyslexia-related difficulties are behavioral and comprise activities aimed at improving reading by adopting process-based approaches. More precisely, such interventions are inspired by theoretical frameworks focusing on specific reading-related cognitive mechanisms, such as phonological processing (e.g., Shaywitz et al., 2004), temporal-auditory perceptual abilities (e.g., Gaab et al., 2007; Thomson et al., 2013), visuo-spatial attentional abilities (e.g., Franceschini et al., 2012), and grapheme-phoneme association (Saine et al., 2011).

Although the outcome measures employed for assessing the effectiveness of remedial methods for DD are in most cases behavioral (e.g., standardized test measuring reading speed and accuracy, phonological awareness, verbal working memory, rapid automatized naming, school proficiency), few studies measured the neurobiological changes associated with DD intervention. Findings from a meta-analysis by Barquero et al. (2014), which considered studies investigating differences in functional activation following reading intervention, are convergent with neurofunctional models of DD (Pugh et al., 2000; Maisog et al., 2008; Richlan et al., 2011) in identifying a central role of left-lateralized inferior frontal, temporo-parietal, and occipito-temporal dysfunctions. In typically-reading adults, the reading system is dominated by a left-sided network, comprising three circuits (Shaywitz and Shaywitz, 2008): (a) A posterior ventral pathway centered in the inferior occipital-temporal area, engaged in the visual processing and recognition of words (Dehaene and Cohen, 2011); (b) A dorsal posterior region comprising the posterior superior temporal, supramarginal, and angular gyri, which is involved in phonological, orthographic, and semantic processing, and grapheme-phoneme conversion (Price, 2012); (c) An anterior component, located in the inferior-frontal gyrus (IFG), involved in phonological processing and articulatory output (Levy et al., 2008). In DD, an underactivation of both temporo-parietal and occipito-temporal regions have been reported (Richlan, 2012).

The correspondence between the improved behavioral outcomes and the neurofunctional reorganization following treatment has, quite recently, led to the hypothesis of a neuromodulatory remedial intervention for DD (Krause and Cohen Kadosh, 2013; Vicario and Nitsche, 2013). To date, few experimental studies have explored the possibility to modulate reading performance using non-invasive brain stimulation (NIBS) by inducing excitability alterations in the brain regions shown to be underactivated in poor readers and individuals with DD.

The present review includes a collection of the studies which have employed transcranial electrical stimulation (tES) aiming at improving reading abilities in both typical readers and individuals with DD. In order to draw preliminary conclusions on the efficacy of neuromodulation as a potential remedial tool for reading difficulties, this review focuses on tES, and specifically transcranial direct current stimulation (tDCS), thus excluding studies employing transcranial magnetic stimulation (TMS) (e.g., Costanzo et al., 2012, 2013). In tDCS, weak direct electrical currents, ranging from 1 to 2 mA, are applied for a short duration (up to 20 min) via two or more electrodes placed on the scalp (Priori et al., 1998; Nitsche and Paulus, 2000). Such transcranial application of current induces alterations of resting membrane potential and thus variation in the response threshold of the stimulated neurons (see Fertonani and Miniussi, 2017). Unlike TMS, the modifications induced by tES are insufficient to induce action potentials (Creutzfeldt et al., 1962; Bindman et al., 1964). However, the alteration in the threshold response via tES can induce long-lasting cognitive changes (Zaghi et al., 2010). Moreover, relative to TMS, tDCS is associated with fewer and minor adverse side effects (i.e., tingling, itching, burning sensation of the skin under the electrode, and in rare cases nausea and headaches) (Fertonani et al., 2015). Such features make tDCS more suitable for a cognitive enhancement program, which require multiple training sessions in order to be effective. For all the aforementioned reasons, the present review focuses on tDCS studies only.

## Aims and methods

The aim of the present review is to explore the effects of different neuromodulation protocols on reading, specifically considering the variability in targeted cortical areas (electrode montages), number of sessions (single-session vs. multiple applications to the same cortical site with the same stimulation polarity), simultaneous application of behavioral interventions (tDCS only vs. tDCS combined with a behavioral intervention), and population targeted (typical readers vs. individuals with DD; children vs. adults). To do so, we performed a systematic review following the PRISMA guidelines (Liberati et al., 2009).

A literature search was conducted on studies published between 2008 and 2018 on Science Direct database using the search string “tDCS” OR “trascranial direct current stimulation” AND “reading” AND/OR “dyslexia.” Furthermore, we searched for additional references in retrieved articles and reviews and checked each article according to our inclusion criteria.

Only the articles meeting the following eligibility criteria have been selected: (a) peer-reviewed publications written in English; (b) studies including reading outcomes; (c) papers providing details of the protocol implemented; (e) presence of a control group or control condition (sham or opposite stimulation polarity).

## Results

**Figure 1:**
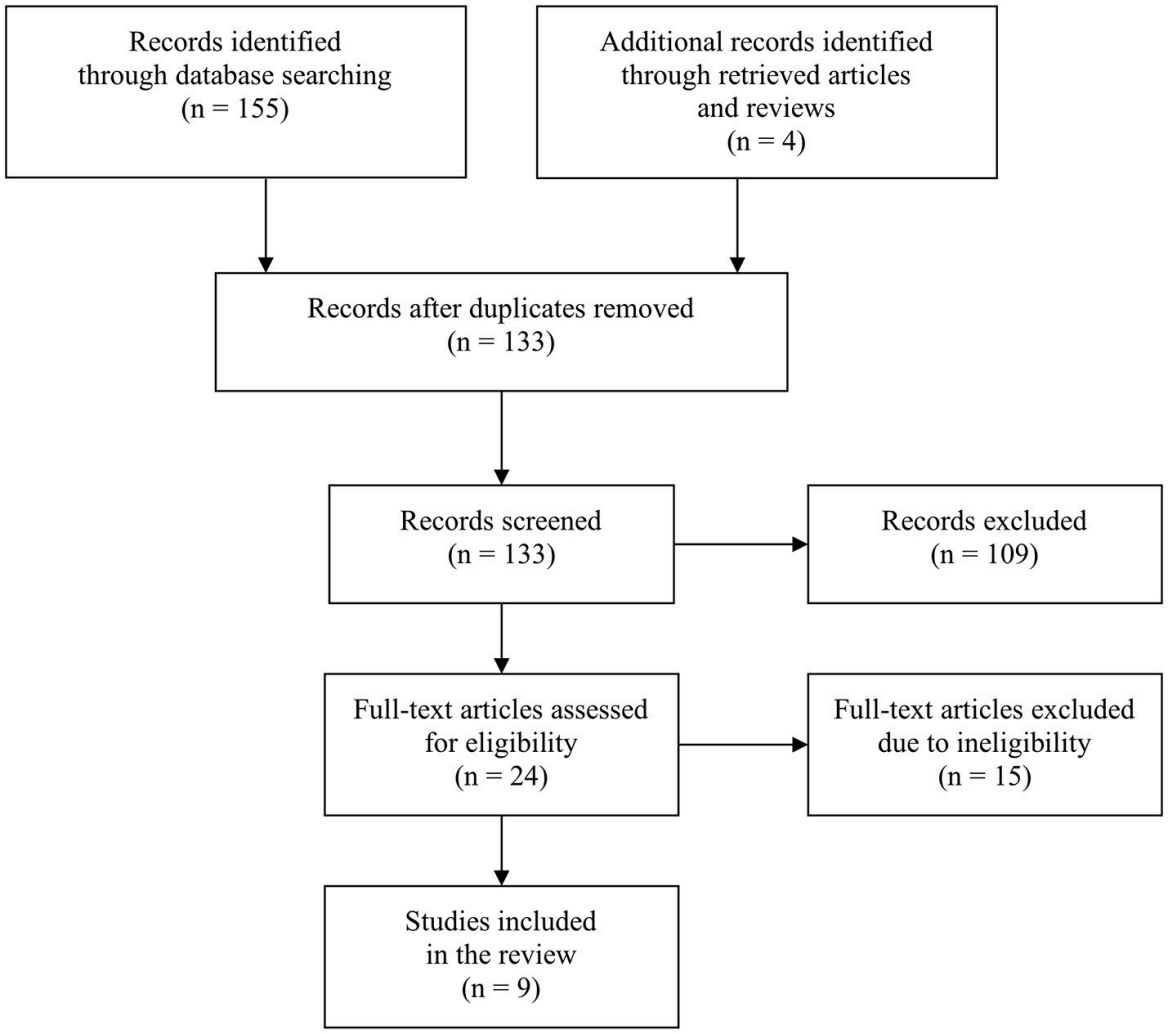
PRISMA flowchart of database search strategy.

One hundred fifty-five records were obtained through database and retrieval articles and reviews searching. However, 135 references were excluded after duplicate removal and title and abstract screening, and another 15 references were further excluded following a full-text assessment (Figure 1). The remaining 9 articles were included in the review (for an overview, see Table 1).

**Table 1:** tDCS studies on reading.

	**Language**	**Design**	**Participants (N, diagnosis, age group)**	**Electrode montage**	**Control condition/group**	**tDCS (mA, density)**	**Sessions (N, duration)**	**Reading outcome measure**	**Reading improvement vs. control condition/group**
^*^Turkeltaub et al., 2012	English (US)	WI	*N* = 25 Typically-reading adults	Left anodal/right cathodal tDCS over pTC	Sham	1.5 mA, 0.06 mA/cm^2^	1 session (20 min)	Word reading efficiency	>
Boehringer et al., 2013	German	WI	*N* = 40 Typically-reading adults	Right cathodal over cerebellum, reference on the right musculus buccinator	Sham	2 mA, 0.08 mA/cm^2^	1 session (25 min)	Word reading speed of color word	=
Thomson et al., 2015	English (US)	MFD	*N* = 39 Typically-reading adults	*N* = 19 Left anodal over TPJ (CP5), reference on controlateral mastoid	*N* = 20 Right anodal over TPJ (CP6), reference on controlateral mastoid	2 mA, 0.06 mA/cm^2^	1 session (20 min)	Word reading efficiency	<
								Non-word reading efficiency	=
				*N* = 19 Left cathodal over TPJ (CP5), reference on controlateral mastoid	*N = 20* Right cathodal over TPJ (CP6), reference on controlateral mastoid			Word reading efficiency	=
								Non-word reading efficiency	=
^†^Heth and Lavidor, 2015	Hebrew	BTW	*N* = 19 Adults with DD	Left anodal over V5 area, reference on controlateral orbito-frontal cortex	Sham	1.5 mA, 0.06 mA/cm^2^	5 sessions (duration N/A)	Text reading speed	>
								Text reading accuracy	=
Costanzo et al., 2016a	Italian	WI	*N* = 19 Children and adolescents with DD	Left anodal/right cathodal tDCS over TP	Right anodal/left cathodal tDCS over TP	1 mA, 0.04 mA/cm^2^	1 session (20min)	Word reading efficiency	=
								Non-word reading efficiency	=
								Text reading accuracy	>
								Text reading speed	=
					Sham			Word reading efficiency	=
								Non-word reading efficiency	=
								Text reading accuracy	>
								Text reading speed	=
^‡^Costanzo et al., 2016b	Italian	BTW	*N* = 18 Children and adolescents with DD	Left anodal/right cathodal tDCS over TP	Sham	1 mA, 0.04 mA/cm^2^	18 session (20min)	Text reading efficiency	=
								High frequency word reading efficiency	=
								Low freq. word reading accuracy	>
								Low freq. word reading speed	=
								Non-word reading accuracy	=
								Non-word reading speed	>
Younger et al., 2016	English (US)	BTW	*N* = 32 Below-average reading adults	Left anodal over IPL (P3), reference on controlateral orbito-frontal cortex	Sham	1.5, 0.06 mA/cm^2^	1 session (20 min)	Word reading efficiency	>
					Right anodal over SPL (CP4), reference on controlateral orbito-frontal cortex				>
Westwood et al., 2017	English (UK)	MFD	*N* = 63 Typically-reading adults	*N* = 20 Left anodal over IFG, reference on controlateral orbito-frontal cortex	*N* = 20 Sham	1.5, 0.06 mA/cm^2^	1 session (25 min)	Word reading speed	=
				*N* = 18 Left anodal over pMTG, reference on contralateral cheek	*N* = 18 Sham			Word reading speed	=
^‡^Costanzo et al., 2018	Italian	BTW	*N* = 26 Children and adolescents with DD	Left anodal/right cathodal tDCS over TP	Sham	1 mA, 0.04 mA/cm^2^	18 session (20 min)	Text reading efficiency	=
								High frequency word reading efficiency	=
								Low freq. word reading efficiency	>
								Non-word reading efficiency	>

*For each study, findings are summarized using symbols representing whether the intervention condition/group induced significant reading improvement, relative to the control condition/group (>), or no differences between conditions/groups emerged (=), or the control condition/group induced significantly higher reading improvement than the intervention condition/group (<) (WI, within; BTW, between; MFD, mixed factorial design)*.

* *During tDCS, participants performed either a phoneme perception task or a color perception task*.

† *The significant improvement reported in the table refers to pre vs. follow-up reading measure (1 week after the end of the intervention)*.

‡ *tDCS was applied in conjunction with a cognitive reading training, in both groups*.

**The significant improvement reported in the table refers to pre vs. follow-up reading measure (1 month and 6 months after the end of the intervention).

### Effect of tDCS on reading in typical-readers

In order to investigate the role of the left posterior temporal cortex (pTC) in reading ability, Turkeltaub et al. (2012) carried out an empirical investigation on healthy adults. The authors designed a tDCS intervention procedure based on functional neuroimaging evidence suggesting reduced left pTC activity in individuals with DD (Maisog et al., 2008) and increased left pTC lateralization in children with DD after successful remedial training (Simos et al., 2002). They hypothesized that enhancing left lateralization of pTC would facilitate lexical access and phonological processing, thus ultimately improving reading efficiency. In a within-subject design, 25 right-handed typically-reading adults underwent two tDCS sessions, on different days, to compare a single-session tDCS intervention with a sham control condition. Criteria for participant's selection included: at least 12 years of education, no history of neurologic, psychiatric disorder, significant head trauma, hearing loss, or personal or family history of learning disorder (including DD). In real tDCS, a constant current of 1.5 mA was applied for 20 min via a pair of 25 cm^2^ electrodes. Such stimulation parameters, resulting in a current density of 0.06 mA/cm^2^, were employed in the majority of the studies included in the present review. As for the electrode montage, the anodal electrode was positioned over the left pTC (midway between T7 and TP7) whereas the cathodal electrode over the contralateral homolog site (midway between T8 and TP8). During the last 15 min of tDCS, participants performed either a phoneme perception task or a color perception task to maintain attention and arousal. Word and non-word reading efficiency was assessed offline, immediately after each session, using the Test of Word Reading Efficiency - TOWRE (Torgesen et al., 1999), in which participants are asked to read aloud lists of words (Sight Word Efficiency subtest) and non-words (Phonetic Decoding Efficiency subtest) as quickly as possible. For both subtests (words and non-words reading), standard score is determined by the number of verbal stimuli read correctly within 45 s. Authors found a significant effect of real tDCS on word reading efficiency performance, compared to the sham condition, and thus confirmed the short-term efficacy of the enhancement of left pTC lateralization via tDCS after just one session. The beneficial effect was specifically driven by a below-average reading subgroup of participants (*N* = 12), namely participants who scored below average (i.e., < 100) in the word reading test in the post-sham assessment. Conversely, the authors did not find a significant effect of real-tDCS in above-average participants (i.e., TOWRE word reading score > 100), thereby supporting the possibility to successfully address this intervention to individuals with reading difficulties.

A later tDCS study by Thomson et al. (2015) failed to replicate the findings of Turkeltaub and colleagues, despite testing a similar intervention protocol. The researchers used tDCS to stimulate an overlapping but slightly superior region, namely the left temporo-parietal junction (TPJ) (CP5). The aim of the study was to further investigate whether the effect found by Turkeltaub and colleagues was the result of the left anodal stimulation or of the conjunction of left anodal and right cathodal stimulation. To do so, Thomson and colleagues implemented a mixed factorial design, which included both a within-subject comparison of the stimulation polarity (i.e., anodal vs. cathodal) and a between-subject comparison of the stimulation hemispheric lateralization (i.e., left CP5 vs. right CP6). No sham condition was included. Thirty-nine right-handed healthy adults were assigned to either a left or right stimulation condition and received, during two separate sessions, anodal or cathodal stimulation (2 mA for 20 min) over TPJ, while the reference electrode was positioned on the contralateral mastoid. Participants had no history or presence of reading disability or any neurological or psychiatric disorder and were not taking any central nervous system-active drugs or medications. Word and non-word reading efficiency measures (Sight Word Efficiency and Phonetic Decoding efficiency subtests of the Test of Word Reading Efficiency - 2nd edition: Torgesen et al., 2011) were administered before each tDCS session and after 10 min from the beginning of the stimulation. Differently than expected by the authors, results showed a mild but significant increase of word reading efficiency following right hemisphere anodal stimulation, compared to left hemisphere anodal stimulation. Moreover, word reading performance decrement was induced by left anodal stimulation. Thomson and colleagues speculated that, since participants were typical readers, no further improvement could yield from the enhancement of the already functioning left hemispheric phonological system. Conversely, they suggested that the activation of the normally less involved right temporo-parietal region could have led to reading improvement, similarly to what was observed in other processing domains (i.e., motor functioning: Boggio et al., 2006). Finally, the absence of a cathodal flow directly at the right temporal parietal junction was proposed as a further explanation for the inconsistent results compared to Turkeltaub et al. (2012).

In their experimental investigation, Younger et al. (2016) targeted a more superior portion of the left temporo-parietal cortex, namely the inferior parietal lobe (IPL), based on its role in smaller-grained grapheme-to-phoneme mapping and its involvement in initial development of the reading network (Pugh et al., 2000). Word reading efficiency of 32 right-handed, low-to-average reading skilled adults (baseline: 80–100 standard score in the Sight Word Efficiency subtest of the Test of Word Reading Efficiency: Torgesen et al., 1999) was measured before and immediately after a single-session tDCS intervention. Inclusion criteria, along with a below-average word reading performance, included no history of neurological disorder, psychiatric disorder, significant head trauma, hearing loss, substance abuse, seizure or migraine, metal implants, and current pregnancy. Participants have been assigned to one of three conditions: anodal tDCS over left IPL, right tDCS over right SPL, or sham. In all conditions the cathode electrode was positioned over the contralateral supraorbital frontal region, so to selectively measure the effect of the anodal stimulation on the target region. The real tDCS parameters replicated the ones used by Turkeltaub et al. (2012) 1.5 mA for 20 min. Results supported the initial hypothesis: Participants who received the anodal stimulation over left IPL showed significantly greater improvement in word reading efficiency, relative to the participants assigned to sham condition and the right anodal over SPL condition (for the latter, the difference trended toward significance). The effect of the montage employed in Younger and colleagues' study resulted in a greater effect size compared to Turkeltaub and colleagues' (Cohen's d: 1.57 vs. 0.46).

More recently, Westwood et al. (2017) used naming and reading tasks to assess the effect of tDCS on the semantic interference effect in word retrieval (e.g., Belke, 2013), namely, the slower and less accurate responses in retrieving a target word when semantically related words are presented. More precisely, authors contrasted stimulation of frontal and temporal areas hypothesizing that frontal stimulation would boost selection mechanisms, thereby reducing the interference effects, whereas temporal stimulation would increase the activation of competing items resulting in a stronger interference, as suggested by Pisoni et al., 2012). Word reading served as a control task, to verify the specificity of semantic interference effect on naming. According to the authors, reading should not be affected by lexical-semantic selection, since orthographic processing is primarily involved (see Belke, 2013). Sixty-three right-handed healthy undergraduate students took part in two 25-min sessions one week apart, during which they completed both reading and picture naming tasks (the order of task presentation was counterbalanced). As for the reading task, 165 semantically related and unrelated words, corresponding to the stimuli of the picture naming task, were presented on a computer screen. Speed and accuracy performance were recorded. However, authors did not analyze error rates for word reading, since they were < 5%. Participants with language impairments, history of migraine, headaches, skin disorders, any adverse experience to previous tDCS, any history of epilepsy or stroke, head/metal implants, any neurological disorders, as well as any volunteers who had participated in a tDCS or TMS study in the previous 6 months, were excluded. Authors implemented a mixed-factorial design, in which participants were assigned to either a frontal stimulation condition (*N* = 20) or a temporal stimulation condition (*N* = 18). In both experimental conditions participants received real and sham tDCS, each in one of the two sessions. Stimulation was delivered for 25 min at 1.5 mA using 25 cm^2^ electrodes. Both picture naming and word reading tasks were completed during the stimulation. In the frontal stimulation condition, the active electrode was placed over the left inferior frontal gyrus (IFG) and the reference electrode (35 cm^2^) was placed over the contralateral supraorbital area, whereas in the temporal stimulation condition the active electrode was placed over the left mid-posterior temporal lobe area (pMTG), and the reference was placed over the contralateral cheek, so to avoid current flow through frontal areas. To control for random variability between sessions, a control group (*N* = 25) was tested using the same protocol, however without receiving any stimulation. No significant effect of tDCS on either reading or naming was found in any condition, as well as no effect of stimulation site (frontal vs. temporal). In light of such findings, authors questioned the reliability of tDCS in inducing cognitive effects in healthy participants using single-session stimulation, in accordance with a quantitative review on a broad spectrum of cognitive outcome measures (including executive functions, language, and memory) by Horvath et al., 2015).

In a study aimed at investigating cerebellar contributions to verbal working memory, Boehringer et al. (2013) tested the effect of cathodal tDCS over the right cerebellum on forward and backward digit spans and other control tasks, among which word reading speed. In such reading task, the time needed to read aloud 42 color words was measured. Fourty right-handed, native German speaking, healthy participants were invited to participate in two tDCS sessions, at least 5 days apart; In each one, they received either cathodal (2 mA for 20 min, using 25 cm^2^ electrodes) or sham tDCS over the right cerebellum (2 cm below the inion and 1 cm posterior to the right mastoid), with the anode placed over the right musculus buccinator. Outcome measures (i.e., forward and backward digit spans, reading of color words, a visually cued sensory-motor task, and finger tapping) were collected before and immediately after tDCS. Authors found that whilst real cathodal tDCS significantly reduced verbal working memory performance, it did not affect word reading.

### Effect of tDCS on reading in individuals with DD

The first tDCS study involving adults with a diagnosis of DD was carried out by Heth and Lavidor (2015). The authors targeted the visual extrastriate area V5/MT, whose activity has been reported to be reduced in individuals with DD (Demb et al., 1998; Eden and Zeffiro, 1998). To identify such stimulation site, the authors adopted a visuo-attention approach and specifically referred to the magnocellular deficit theory of DD (Stein, 2012). According to this theory, DD is associated with an abnormal visual motion processing, due to a dysfunction of the magnocellular system, a perceptual pathway projecting from the lateral geniculate nucleus to primary visual areas, responsible for detecting contrast, motion, and rapid changes in the visual field. As the hypothesis of causal role of the magnocellular system in DD is highly controversial due to insufficient empirical support and contrasting findings (Amitay et al., 2002; Ramus et al., 2003), the visual magnocellular dysfunction has been interpreted, instead, as a consequence of impoverished reading (Olulade et al., 2013). To examine the magnocellular involvement in the reading process, Heth and Lavidor designed an intervention comprising five tDCS sessions over 2 weeks, in which anodal stimulation was applied over the V5 area (1.5 mA for 20 min), with the right orbito-frontal cortex as a reference site. Nineteen right-handed, native Hebrew speaking adults who had previously received a diagnosis of DD, without a comorbidity with attention deficit and hyperactive disorder (ADHD) nor neurological or psychiatric conditions, were randomly assigned to either the anodal or a sham condition. Text reading speed and accuracy were assessed before, immediately after and a week after the end of the 5-session intervention. Three one-page-long passages at 9th grade level, which are routinely included in the DD diagnostic procedure in Israel (Tov-Li, 1999), were used to assess participants' text reading speed and accuracy. The anodal tDCS group showed a significant improvement in text reading speed at follow-up assessment, compared to the sham group, whereas no difference between condition occurred immediately after the end of the intervention. Reading accuracy did not improve at any time point. These findings were interpreted by the researchers as indication of the involvement of the V5 area in reading.

Costanzo et al. (2016a), Costanzo et al. (2016b), and Costanzo et al. (2018) were the first to study the effect of tDCS on young populations with DD. Several researchers called for caution in the application of NIBS to children, pointing out the unknown consequences and the possible side effects of stimulating a developing brain (Kadosh et al., 2012; Krause and Cohen Kadosh, 2013). The major concern regards the potential deterioration of certain abilities as a consequence of the enhancement of specific learning skills. To date, empirical evidence from the application of tDCS to developmental samples is still limited (Mattai et al., 2011; Schneider and Hopp, 2011; Auvichayapat et al., 2013; Amatachaya et al., 2015) and no safety guidelines for children as been yet established. Despite these concerns, Costanzo and colleagues stressed the importance of the exploration of such potentially effective intervention for DD in developmental age, which could be critical to foster school learning and, consequentially, broaden future occupational opportunities.

In the light of the contrasting findings from the previous studies (Turkeltaub et al., 2012; Thomson et al., 2015), Costanzo and colleagues explored the optimal polarity of the stimulation for children and adolescents with DD in a single-session intervention study (Costanzo et al., 2016b). A within-subject design was implemented to compare left and right anodal stimulation over the temporo-parietal region: Midway between P7 and TP7 and midway between P8 and TP8, respectively. The reference electrode was placed on the contralateral homologs site in both conditions, in order to exclude brain regions involved in the reading process, such as the prefrontal and the occipital cortices (Eckert, 2004; Richlan, 2014) and thus focus on the role of temporo-parietal regions. Slightly lower current intensity was used (1 mA) and the stimulation was delivered for 20 min. A sample of 19 right-handed, native Italian speaking children and adolescents with a diagnosis of DD, aged 10-18 years, participated in three tDCS sessions (i.e., left anodal/right cathodal, right anodal/left cathodal, and sham) on different days. Measures of word reading (20 high-frequency words and 20 low-frequency words), non-word reading (20 non-words), and text reading (a 400-syllable long passage) were collected before and immediately after each tDCS session. Reading accuracy was expressed by number of errors (1 point was assigned for each letter substitution and 0.5 point for every self-correction or hesitation), whereas reading speed by total reading time (in seconds). Results showed a significant text reading accuracy improvement following left anodal/right cathodal tDCS and an increase in errors after left cathodal/right anodal tDCS, relative to the other conditions. These findings, which are consistent with those of Turkeltaub and colleagues, support the efficacy of the simultaneous action of left anodal and right cathodal tDCS in inducing reading improvement in children and adolescents with DD.

In a second study by the same authors (Costanzo et al., 2016a), the tDCS protocol, which was found to be effective in the previous exploration (Costanzo et al., 2016b) (i.e., left anodal/right cathodal over the temporo-parietal regions), was applied to a group of children and adolescent with DD. To further improve reading abilities, and induce medium-term positive effects, a multiple-session intervention protocol was designed in which tDCS was paired with a remedial cognitive training, comprising tachistoscopic and phonic (training on phoneme awareness and grapheme-phoneme conversion) reading exercises. Eighteen right-handed, native Italian speaking participants were randomly assigned to either a real tDCS (1 mA for 20 min) or sham condition. Participants had no history of neurological disease, nor a family history of epilepsy, nor comorbidity with ADHD. Both groups participated in an 18-session intervention including the cognitive training over 6 weeks. The same reading measures as the previous study (Costanzo et al., 2016b) were collected before, immediately after, and 1 month after the end of the treatment. Consistently with the previous study, the active tDCS groups showed significant improvements in low frequency reading accuracy and non-word reading speed, compared to the sham group. Furthermore, the improvements persisted a month after the end of the intervention. Performance increases were specifically found in reading tasks involving phonological processing and letter-sound mapping (i.e., low frequency word and non-word reading).

In a more recent study, the same authors (Costanzo et al., 2018) replicated the same protocol on a larger group of children and adolescents with DD, including a further follow-up assessment 6 months after the end of the intervention, so to measure its long-term efficacy. Twenty-six right-handed children and adolescents with a diagnosis of DD were selected on the basis of the same inclusion criteria as the previous investigation. Differently than the previous study, results were reported considering a reading efficiency index, thus representing speed and accuracy together, for each task (i.e., high-frequency word, low-frequency word, non-word, and text reading). Whereas the participants who received sham tDCS (*N* = 13) did not show reading changes at any time point, the experimental group (*N* = 13) showed significant improvements in low-frequency word reading (1- and 6-month after the end of the treatment) and in non-word reading (immediately after, 1 month after, and 6 months after the end of the treatment). Costanzo and colleagues interpreted such findings as an evidence of tDCS delayed but long-lasting beneficial effect. Consistently with the previous study, no effects on high-frequency word nor text emerged.

Finally, the protocol of an ongoing study testing the effect of a multiple-session tDCS intervention combined with a cognitive training for DD has been recently reported (Cancer and Antonietti, 2017). A sample of undergraduate students with a diagnosis of DD has been involved in an intervention comprising a novel rhythm-based reading training (Bonacina et al., 2015; Cancer et al., 2016) paired with tDCS for 10 daily sessions over 2 weeks. The left temporo-parietal region was stimulated at a constant current of 1.5 mA for 20 min, and the electrode montage replicated the one used by Costanzo et al. (2016a). Preliminary results from three single cases who took part in the real tDCS condition provided encouraging evidence about the efficacy of the combined intervention on undergraduate students with DD (Cancer and Antonietti, 2017). However, the pattern of reading sub-components improvement seemed to depend greatly on individual reading profile at baseline. Since no conclusion about the role of tDCS on reading can be draw from such preliminary single-case data, the study was not included in the overall comparison.

## Discussion

Despite the limited number of studies included in the present review, and the procedural and methodological dissimilarities between them, a descriptive and critical analysis of their findings could provide some insights into tDCS modulation of reading processes. Characteristics and main results of the nine reviewed studies have been summarized in Table 1.

Considering the different cortical areas targeted by anodal stimulation, the majority of the studies focused on the left temporo-parietal cortex (i.e., pTC, TPJ, pMTG, IPL). Such regions are typically involved in phonological, orthographic, and semantic processing during reading tasks (Price, 2012) and underactive in individuals with DD (see Richlan, 2012). Significant effects on reading were observed following left anodal temporo-parietal montages in 5 out of 7 studies (Turkeltaub et al., 2012; Costanzo et al., 2016a,b, 2018; Younger et al., 2016).

However, the type of population targeted, specifically typical readers vs. below-average readers and individuals with DD, appeared to significantly account for the outcomes of temporo-parietal anodal tDCS interventions. As suggested by Thomson et al. (2015), whose findings on healthy participants showed an opposite trend relative to the other interventions, individuals with poor reading skills are more suitable to benefit from a neuromodulatory intervention enhancing left temporo-parietal lateralization, due to anomalies in their cortical activity, whereas similar beneficial effects cannot be replicated in already proficient readers. Null effect of a stimulation protocol similar to Thomson and colleagues' on healthy adults were also reported by Westwood et al. (2017). Consistently, the ameliorative effects of a similar protocol reported by Turkeltaub et al. (2012) were driven by a below-average reading subgroup of participants, whilst no significant effect of tDCS was found in above-average participants. As suggested by Westwood et al. (2017), tDCS modulatory mechanisms are most likely to induce effect on cognition in brains with low or dysfunctional neuronal excitability, rather than already close to an optimal level of excitability.

The association between the precise electrode placement (i.e., anodal tDCS over superior vs. inferior portion of the left TP cortex; unilateral vs. bilateral montages) and the modulation of a specific reading sub-processes (i.e., grapheme-to-phoneme mapping vs. lexical representation access) was not consistent across studies. Similar left anodal/right cathodal montages (Turkeltaub et al., 2012; Costanzo et al., 2016a,b, 2018; Younger et al., 2016) led to improvement in different reading outcomes (either word efficiency, text accuracy, or low frequency and non-word reading speed). Costanzo et al. (2016a) suggested that, in order to enhance word and text reading, the medium and inferior temporal gyri, which are specifically involved in whole-word recognition (Dehaene et al., 2005; Vinckier et al., 2007), should be targeted. Consistently, the left temporo-parietal dysfunction in adults with DD has been observed during the performance of phonological tasks, such as non-word reading, phonological lexical decision, and word rhyme judgment (for a meta-analysis, see Richlan et al., 2011), whilst underactivation of the left fusiform gyrus (Brambati et al., 2006) and of occipito-temporal regions (McCrory et al., 2005) was specifically associated with word reading. Whereas this suggestion would explain why Costanzo and colleagues' left temporo-parietal anodal stimulation protocols did not induce changes in word reading, it would not explain why in other studies on below-average-reading adults word reading was improved using similar tDCS interventions. Specifically, word reading efficiency was successfully modulated by anodal stimulation of left temporo-parietal regions in below-average readers, as seen in Turkeltaub et al. (2012) and Younger et al. (2016) studies.

We suggest that compensatory rather than “normalizing” functional changes could have enhanced reading ability in adult with poor reading skills via the recruitment of alternate circuits for word reading. According to such hypothesis, below-average adult readers, after tDCS modulation of the temporo-parietal areas, would rely on grapheme-to-phoneme mappings for word reading, instead of increasing the functionality of the circuits normally activated in adult proficient readers. However, only older populations with reading difficulties would exhibit such compensatory changes following intervention, whereas children with DD would exhibit normalization changes. The hypothesis is consistent with neuroimaging evidence showing an increased activation in both left middle temporal and posterior superior temporal areas after a successful behavioral treatment in children with DD, which improved both word and non-word reading (Simos et al., 2002; Barquero et al., 2014). On the other hand, a study measuring the neurofunctional and behavioral changes in adults with DD after an intensive phonology-based intervention program found significant increases in left hemisphere inferior parietal lobule and intraparietal sulcus in correspondence of both non-word and text reading, thus showing that improved phonological processing was transferred to other aspects of reading ability as well (Eden et al., 2004).

As for the electrode position differences in studies targeting difference portions of the temporo-parietal cortex, we argue that no major outcome variability was accounted for by it. Due to its limited spatial resolution, tDCS is not suitable for stimulating focal portions of the cortical tissues and the current will most likely flow outwards the targeted site, thus affecting the surrounding areas.

The ameliorative effects of bilateral tDCS montages reported by Turkeltaub et al. (2012), Costanzo et al. (2016a), Costanzo et al. (2016b), and Costanzo et al. (2018), in which cathodal stimulation was applied to right temporo-parietal regions, are consistent with literature on children with DD showing a reduction of right temporal activation after a successful reading intervention (Shaywitz et al., 2004) and a greater activation of the same area in children with DD who did not show reading improvements after a behavioral intervention (Odegard et al., 2008). Conversely, such effects were not replicated in typical readers, as shown by Thomson et al. (2015), who found a positive effect of right temporo-parietal anodal stimulation on word reading.

Only three studies included in the present review measured the effect of anodal tDCS outside the temporo-parietal cortex. Among these, null effect of tDCS intervention were found in studies on typical readers (Boehringer et al., 2013; Westwood et al., 2017), in spite of the site of anodal stimulation (i.e., cerebellum or inferior frontal gyrus). In contrast, Heth and Lavidor (2015) found positive effect of anodal stimulation over an occipital visual area (V5/MT) in adults with DD, as measured by text reading speed improvement 1 week after the end of the intervention. Such findings are consistent with the underactivation of occipital and occipito-temporal regions, specifically involved in visual processing and recognition of word, which were found in adults with DD (Richlan, 2012). Therefore, it will be of interest to further investigate its role on word reading as well.

Interestingly, the comparison between single- (Turkeltaub et al., 2012; Thomson et al., 2015; Costanzo et al., 2016b; Younger et al., 2016) and multiple-session tDCS interventions (Heth and Lavidor, 2015; Costanzo et al., 2016a, 2018) did not appear to give reason for major variability in the results. However, the small number of studies testing repeated tDCS methodologies for improving reading does not allow to draw any general conclusion.

Only two studies, by the same authors and with the same protocol (Costanzo et al., 2016a, 2018), tested the efficacy of the simultaneous application of behavioral intervention targeting reading during tDCS. Such approach, associated with a multiple-session protocol, was the only one to be effective in inducing significant improvements in reading measures involving phonological processing and grapheme-to-phoneme mapping. Furthermore, these studies also provided evidence of mid-term (up to 1 month) and long-term (up to 6 months) efficacy of left temporo-parietal tDCS on reading.

Finally, three studies conducted on younger populations with DD (Costanzo et al., 2016a,b, 2018) confirmed the possibility of successfully employing tDCS as a remedial intervention for DD, especially when combined with a cognitive training targeting reading. These pioneering investigations showed that repeated tDCS applications can be tolerated by children and adolescents, without significant discomfort or adverse effects reported up to 3 months after the end of the intervention.

## Conclusions

The collection of studies included in the present descriptive and critical review supported the hypothesis to use neuromodulation for improving reading skills in individuals with DD. Positive effects have been reported in the majority of the tDCS studies reviewed. Anodal tDCS over left temporo-parietal region was the most frequently investigated montage, which was shown to be successful in improving reading, when compared to alternative tDCS montages. However, the exact nature of the observed reading improvements was rather controversial. Enhancement of differential sub-processes of the reading ability (i.e., grapheme-to-phoneme mapping or lexical access; reading speed vs. reading efficiency) yielded from similar stimulation procedures. Furthermore, the outcome of the intervention varied according to the population targeted: tDCS was found to be specifically effective in poor readers and individuals with DD rather than typical readers.

Overall, tDCS appears to be a promising remedial tool for reading difficulties, even when applied to younger populations. However, further empirical evidence is needed to confirm its potential as a successful intervention method for DD.

As a future direction, reading performance gains should be maximized by combining specific approaches to reading remediation with cortical neuromodulatory techniques, so to engage specific reading sub-processes via neuroplasticity increase (Vicario and Nitsche, 2013). Furthermore, learning paradigms comprising repeated cortical stimulation applications resulting in cumulative effects could provide a medium- to long-term efficacy of the intervention.

## Author contributions

AC and AA contributed to the conception of the work. AC was responsible for the literature search, selection of the articles, and manuscript drafting, the critical revision of the article was shared by both authors. AC and AA gave their final approval of the version to be published.

### Conflict of interest statement

The authors declare that the research was conducted in the absence of any commercial or financial relationships that could be construed as a potential conflict of interest. The reviewer IC and handling Editor declared their shared affiliation, at the time of the review.
